# Identification and characterization of mycoviruses in transcriptomes from the fungal family ceratocystidaceae

**DOI:** 10.1007/s11262-024-02112-4

**Published:** 2024-10-08

**Authors:** Bianca Hough, Brenda Wingfield, David Read

**Affiliations:** Forestry & Agricultural Biotechnology Institute (FABI), Department of Biochemistry, Genetics & Microbiology, University of Pretoria, Pretoria, South Africa

**Keywords:** Mycoviruses, Ceratocystidaceae, Mitoviruses, *Thielaviopsis*, *Ceratocystis*

## Abstract

Mycoviruses pervade the fungal kingdom, yet their diversity within various fungal families and genera remains largely unexplored. In this study, 10 publicly available fungal transcriptomes from Ceratocystidaceae were analyzed for the presence of mycoviruses. Despite mycovirus associations being known in only four members of this family, our investigation unveiled the discovery of six novel mycoviruses. The majority of these mycoviruses are composed of positive sense single stranded RNA and are putatively assigned to the viral family *Mitoviridae* (with tentative classification into the genera *Unuamitoviru*s and *Duamitovirus*). The double stranded RNA viruses, however, were associated with the family *Totiviridae* (with tentative classification into the genus *Victorivirus*). This study also revealed the discovery of an identical unuamitovirus in the fungal species *Thielaviopsis ethacetica* and *Thielaviopsis paradoxa*. This discovery was notable as these fungal isolates originated from distinct geographical locations, highlighting potential implications for the transmission of this mitovirus. Moreover, this investigation significantly expands the known host range for mycoviruses in this family, marking the initial identification of mycoviruses within *Ceratocystis platani*, *Thielaviopsis paradoxa*, *Thielaviopsis ethacetica,* and *Huntiella omanensis*. Future research should focus on determining the effects that these mycoviruses might have on their fungal hosts.

**Supplementary Information:**

The online version contains supplementary material available at 10.1007/s11262-024-02112-4.

## Introduction

Viruses are among the most abundant and diverse biological entities found on Earth, capable of infecting a wide range of life forms, including animals, plants, bacteria, protists, and fungi [1–5]. The study of viruses is of paramount importance in the context of managing and preventing viral disease, understanding viral evolution and ecological impact, and harnessing their potential in therapeutics and biotechnology [6–9]. However, the majority of virus-related research has primarily focused on economically significant plant and animal viruses, often neglecting other important viruses, such as fungal viruses (mycoviruses).

Mycoviruses are prevalent throughout the fungal kingdom. They have been found in most of the major fungal taxonomic groups, including some of the early diverging lineages such as Mucoromycota and Zoopagomycota [10, 11]. They also exhibit great diversity, and have been classified into 23 recognized viral families and one unclassified genus by the International Committee on the Taxonomy of Viruses (ICTV) [12]. Classification is based on multiple factors, such as genome characteristics (e.g., nucleic acid type, genome orientation, length, and organization), host specificity, and percent identity to closely related viruses [12]. The majority of mycoviruses are composed of double stranded RNA (dsRNA) or single stranded RNA (ssRNA), but a few with single stranded DNA genomes have also been discovered [11]. For example, members of the species *Gemycircularvirus sclero1* and *Gemytripvirus fugra1* belong to the family *Genomoviridae*, which consists of DNA viruses [13, 14].

Mycoviruses may cause several different effects on their fungal hosts. While the majority of known mycoviruses induce asymptomatic infections, some may also evoke hypervirulence (enhanced virulence) or hypovirulence (reduced virulence) in their respective hosts [15]. In the 1960s, hypovirulence-inducing mycoviruses were recognized as the primary cause of La France disease [16]. This condition had significant detrimental effects on the cultivation of commercially produced mushrooms (*Agaricus bisporus*) [16, 17]. These mushrooms were infected with Agaricus bisporus virus 1 (AbV1), a chrysovirus which was later identified as the primary causal agent of this disease, and displayed reduced mycelial growth, malformed fruiting bodies, and rapid deterioration of these fruiting bodies after harvesting [18, 19]. Within this particular framework, mycoviruses can be perceived as harmful because some can cause economic losses in the production of edible mushrooms [19].

Mycoviruses resulting in fungal disease can also present potential benefits from a biocontrol standpoint, as they have the capacity to reduce the impact of plant pathogenic fungi and thus plant disease [20, 21]. A notable example is the use of members of the species *Alphahypovirus cryphonectriae* (Cryphonectria parasitica hypovirus 1; CHV-1) in the control of the chestnut blight pathogen, *Cryphonectria parasitica*, in certain regions of Europe [22–25]. Consequently, a significant portion of mycovirus-related research has revolved around the discovery and characterization of mycoviruses which are capable of inducing hypovirulence in plant pathogenic fungi [26–29]. This research is also driven by the need for alternatives to chemical fungicides, which poses risks to environmental and human health, and which have become increasingly ineffective due to fungal resistance [30–33].

The rapid advancement in next-generation sequencing (NGS) technologies, coupled with various bioinformatics tools, have significantly increased the discovery of mycoviruses [34–36]. Moreover, this has resulted in an increased availability of fungal transcriptomic datasets on open-access platforms like the National Centre for Biotechnology Information (NCBI) sequencing reads archive (SRA), which enables the identification of mycoviruses within these transcriptomes [37, 38]. However, our current understanding of mycoviral diversity in various fungal genera and families remains inadequate, highlighting the need for further research to obtain a more comprehensive understanding of mycoviral diversity within these groups.

Ceratocystidaceae encompasses a diverse group of fungi, including those with substantial economic impact, such as plant pathogens, insect symbionts, and agents responsible for timber degradation [39–42]. While some members of Ceratocystidaceae have been found to harbor mycoviruses, the knowledge in this regard is limited. Two members of the genus *Endoconidiophora* for example, are known to associate with a partitivirus [43]. Recently, mycoviruses have also been found in publicly available transcriptomes from *Ceratocystis fimbriata* and *Ceratocystis cacaofunesta* [44]. However, a comprehensive understanding of mycoviral diversity within *Ceratocystidaceae* is still lacking, necessitating further research to explore and characterize the full extent of mycoviral diversity in this fungal family.

The aim of this study was to gain a deeper understanding of mycoviral diversity within Ceratocystidaceae. To achieve this, publicly available transcriptomes from members of this family were analyzed for the presence of mycoviruses using bioinformatics approaches. The genomes of any potential mycoviruses were then characterized, and their phylogenetic relationships with closely related viruses were investigated in order to elucidate their taxonomy and evolutionary connections. This study provides a comprehensive and detailed overview of mycoviral diversity within the Ceratocystidaceae family. Furthermore, this study facilitated the identification of mycoviruses that could be further evaluated as potential biocontrol agents in future research.

## Materials and methods

### Fungal transcriptomes

Unassembled transcriptomic datasets from members of Ceratocystidaceae were retrieved from the NCBI SRA database [45], and can be found in Table 1. To ensure data integrity, the quality of these datasets was assessed using FastQC [46] and processed with Trimmomatic (version 0.36) [47], with a minimum Phred score of 33 and 20 for sequences generated with IonTorrent and Illumina platforms, respectively.

**Table 1:** Overview of publicly available fungal transcriptomes used for the discovery of mycoviruses in Ceratocystidaceae

Genus	Isolate	Fungal plant/insect host and origin	SRA ID	Instrument and selection step used
*Ambrosiella*	*Ambrosiella xylebori* CBS 110.61	*Xyleborus compactus* gallery in *Coffea canephora*—Ivory Coast	SRR5865576	Illumina HiSeq 2000—unknown
*Bretziella*	*Bretziella fagacearum* C519	*Quercus rubra—*Unknown origin	SRR13083620	Illumina NovaSeq 6000—unknown
*Ceratocystis*	*Ceratocystis platani* MS580	*Platanus* x *hispanica—*Tuscany, Italy	SRR13858934	Illumina MiSeq—unknown
*Huntiella*	*Huntiella abstrusa* CMW21092	*Eucalyptus*—Teso East, Riau province, Indonesia	SRR22044985 SRR22044986 SRR22044987	Ion Torrent Proton—mRNA enrichment
	*Huntiella omanensis* CMW44442	Lab strain—South Africa	SRR5640136 SRR5640137 SRR5640138	Ion Torrent Proton—mRNA enrichment
	*Huntiella omanensis* CMW44450	Lab strain—South Africa	SRR5639921 SRR5639922 SRR5639923	Ion Torrent Proton—mRNA enrichment
	*Huntiella omanensis* CMW 11056	*Mangifera indica*—Oman	SRR5640139 SRR5640140 SRR5640141	Ion Torrent Proton—mRNA enrichment
	*Huntiella moniliformis* CMW 36919	*Theobroma cacao*—Cameroon	SRR5640143 SRR5640144 SRR5640145 SRR5640146 SRR5640147 SRR5640148	Ion Torrent Proton—mRNA enrichment
*Thielaviopsis*	*Thielaviopsis paradoxa* R-189	*Elaeis guineensis* -Meta, Colombia	SRR15533162	Illumina HiSeq 2000—poly (A) selection
	*Thielaviopsis ethacetica* JCM 6961	*Saccharum officinarum*—Sertãozinho, São Paulo, Brazil	SRR12744487 SRR12744488 SRR12744489 SRR12744490	Illumina HiSeq 2500—poly (A) selection

Fungal isolates representing five genera within the Ceratocystidaceae family were procured from the Sequencing Reads Archive. Each dataset is identified by a distinct SRA ID, which serves as a unique accession number. In cases where a single isolate was associated with multiple datasets, multiple accession numbers are shown. The table also includes information about the fungal plant/insect host, origin, the type of instrument used and, where applicable, the selection step used in library preparation

### Bioinformatics

The trimmed datasets were de novo assembled with rnaviralSPAdes (version 3.15.0) [48]. Following assembly, the contigs were imported into CLC genomics workbench 22 (Qiagen Bioinformatics, Aarhus, Denmark), where they were translated from nucleotide sequences to protein sequences in all possible reading frames with the ‘Translate to Protein’ tool. These sequences were aligned to viral protein sequences within a custom viral protein database, using the BLAST toolkit within CLC genomics workbench. This custom database was created in March 2022, and comprised viral protein sequences from all known viral families containing mycoviruses, as well as unclassified mycoviruses. These protein sequences were obtained from the NCBI protein database and were regularly updated as new viral families and genera were released (Last updated in April 2023). The output from this was manually inspected, and putative mycoviral contigs were selected for downstream analysis based on, alignment length and E-value (< 10^–15^). To differentiate real viral sequences from host sequences, the selected contigs were then subjected to BLAST evaluation against a non-redundant protein sequence database using the NCBI BLASTp tool [49].

### Genomic characterization and phylogenetic analysis of mycoviruses

The open reading frames (ORFs) of all putative mycoviruses were determined with NCBI ORF finder [50]. In instances where putative mycoviruses showed homology to mitoviruses, the mitochondrial genetic code was used to identify ORFs, while the conventional genetic code was used for all other viruses. Where necessary, the web-based version of Mfold [51] was used to predict the secondary 5’ and 3’ UTRs of certain mycoviruses. The web-based version of the tool ProbKnot [52] was also used to predict pseudoknots in the ORF-junction region of putative totiviruses. Subsequently, the conserved protein domains within the mycoviral ORFs were identified using the NCBI BLASTp tool. To perform phylogenetic analyses, the ORFs containing an RNA-dependent RNA polymerase (RdRp) domains were aligned with reference sequences of cognate viral protein sequences obtained from the NCBI GenBank. The ClustalW program in MEGA 11 was used to perform these alignments [53]. Additionally, MEGA 11 was used to determine the best-fit protein model for each alignment. Maximum Likelihood (ML) phylogenetic trees were constructed with IQ-TREE (version 2.1.3), using the best-fit model for the analysis [54]. The RdRp amino acid sequences, and where applicable, the coat proteins (CP), from known mycoviruses and a selection of closely related viruses were aligned using the web-based Clustal Omega tool [55]. This tool was specifically used to create percent identity matrices and used to identify shared motifs in the RdRp protein.

## Results

### Summary of identified viral sequences

From the 10 fungal isolates evaluated in this study, seven were found to be associated with mycoviruses. A total of five mycoviruses possessed positive sense ( +) ssRNA genomes, while two were dsRNA viruses. It should be noted that mycoviral contigs with similarity to viruses from the families *Totiviridae* and *Endornaviridae* were also observed for *H. omanensis* CMW44442 and *C. platani* MS580, respectively. However, these were significantly fragmented, and did not consist of any known domains of proteins. Thus, they were omitted from further analysis. An overview of these mycoviruses and their characteristics can be found in Table 2.

**Table 2:** Taxonomic and genomic overview of putative mycoviruses identified in the transcriptomes of fungi from Ceratocystidaceae

Host	Genome type	Virus Family	Putative virus name	Virus abbrev	Contig Length (nucleotide)	Contig coverage	Protein ID	Accession no
*Ceratocystis platani* MS580	+ ssRNA	*Mitoviridae*	Ceratocystis platani RNA virus 1	CpRV-1	3150	19.78	Mitovir RNA pol	BK065021
^a^*Huntiella omanensis* CMW44450	dsRNA	*Totiviridae*	Huntiella omanensis RNA Virus 1 ORF-2	HoRV-1_ORF-2	2322	9.22	RdRP 4	BK065019
^ab^*Huntiella omanensis* CMW44450	dsRNA	*Totiviridae*	Huntiella omanensis RNA Virus 1 ORF-1	HoRV-1_ORF-1	1852	27.18	Totivirus coat	BK065020
^c^*Thielaviopsis paradoxa* R-189	+ ssRNA	*Mitoviridae*	Thielaviopsis paradoxa RNA virus 1	TpRV-1	2599	339.41	Mitovir RNA pol	BK065014
*Thielaviopsis paradoxa* R-189	dsRNA	*Totiviridae*	Thielaviopsis paradoxa RNA virus 2	TpRV-2	5460	42.42	Totivirus coat RdRP 4	BK065015
^c^Thielaviopsis ethacetica JCM -6961	+ ssRNA	*Mitoviridae*	Thielaviopsis ethacetica RNA virus 1	TeRV-1	2620	2094.42	Mitovir RNA pol	BK065016
Thielaviopsis ethacetica JCM -6961	+ ssRNA	*Mitoviridae*	Thielaviopsis ethacetica RNA virus 2	TeRV-2	2616	147.73	Mitovir RNA pol	BK065017
Thielaviopsis ethacetica JCM -6961	+ ssRNA	*Mitoviridae*	Thielaviopsis ethacetica RNA virus 3	TeRV-3	2991	2145.99	Mitovir RNA pol	BK065018

^a^Indicates truncated contigs.^ab^Indicates viral segments without an RdRp domain, which may belong to other truncated viruses in the assemblies.^c^Indicates viruses which are identical and are likely to be different strains of the same viral species are displayed at the bottom of their respective columns. To enhance readability, totiviruses from this study have been highlighted in red. It is important to note that HoRV-1, due to the absence of a full-length genome and thus a CP, has been excluded from the CP alignments

### Characterization of viruses from mitoviridae

Four putative novel viruses were classified as ( +) ssRNA viruses based on the match found in the top Blastp result. The fungi *C. platani* and *T. paradoxa* were each associated with single mycoviruses from the family *Mitoviridae*, putatively named Ceratocystis platani RNA virus 1 (CpRV-1) and Thielaviopsis paradoxa RNA virus 1 (TpRV-1), respectively. *T. ethacetica* on the other hand, was found to harbor two novel mitoviruses, with the putative names, Thielaviopsis ethacetica RNA virus 2 (TeRV-2), and Thielaviopsis ethacetica RNA virus 3 (TeRV-3). Interestingly, a nearly identical mitovirus to TpRV-1 was also discovered in *Thielaviopsis ethacetica* and was given the putative name Thielaviopsis ethacetica RNA virus 1 (TeRV-1).

**Fig. 1:**
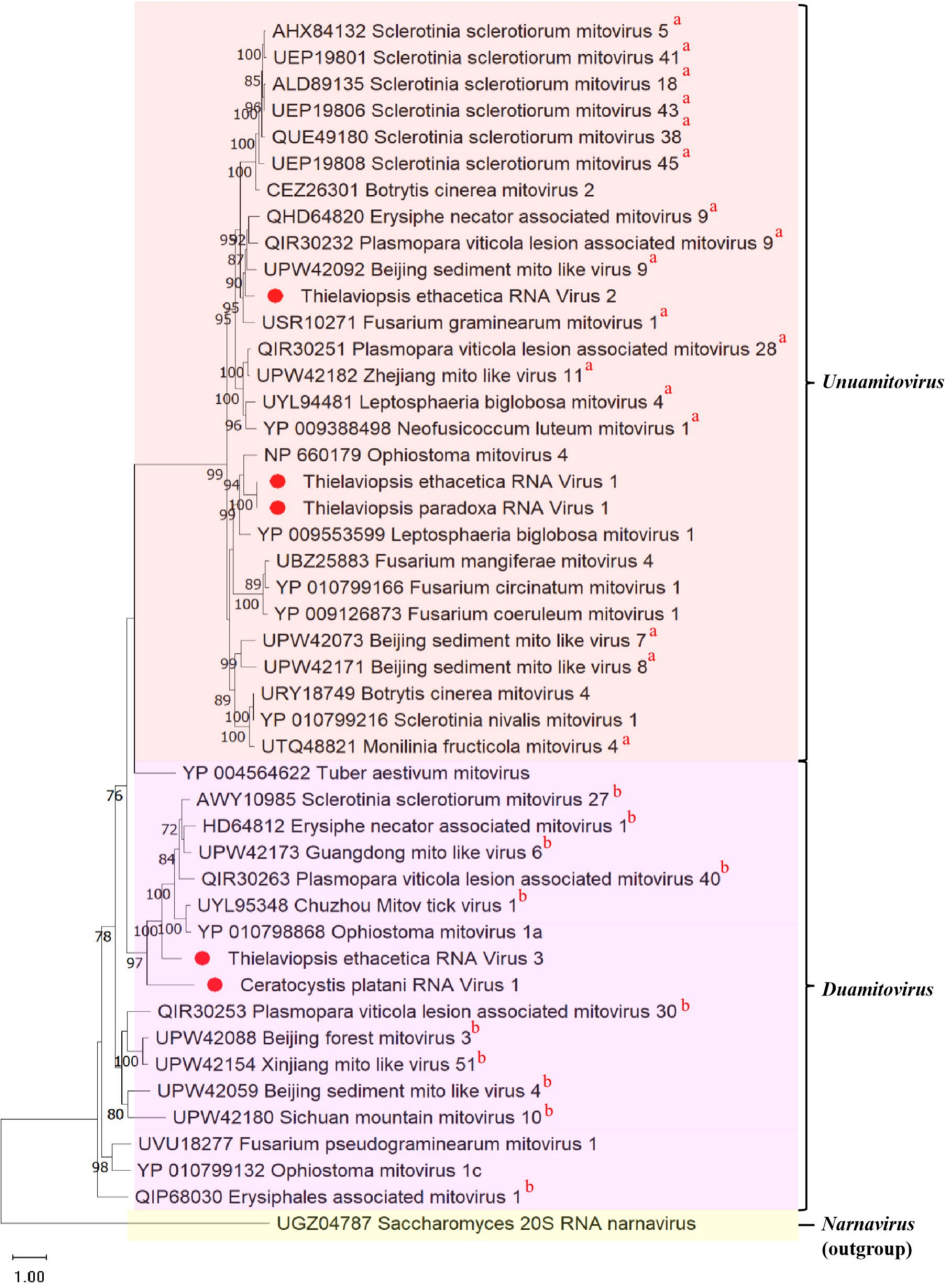
Phylogenetic tree showing maximum likelihood analysis of the RdRp protein sequences from the mitoviruses discovered in this study, with those of related viruses. The phylogeny was generated using IQ-TREE, using the Whelan and Goldman model with frequencies, invariant sites, and gamma distribution. Bootstrapping with 1000 replicates was performed, and corresponding percentages are indicated next to the branches. Only bootstrap values equal to or exceeding 70 are displayed. The Saccharomyces 20S RNA Narnavirus was used as an outgroup. The red circles (●) indicate mycoviruses discovered in this study. **a** Denotes unuamitoviruses and b: duamitoviruses which have not yet been taxonomically assigned by the ICTV (https://ictv.global/taxonomy). These mitoviruses exhibit genetic characteristics and phylogenetic relationships that strongly suggest their classification within these genera

**Fig. 2:**
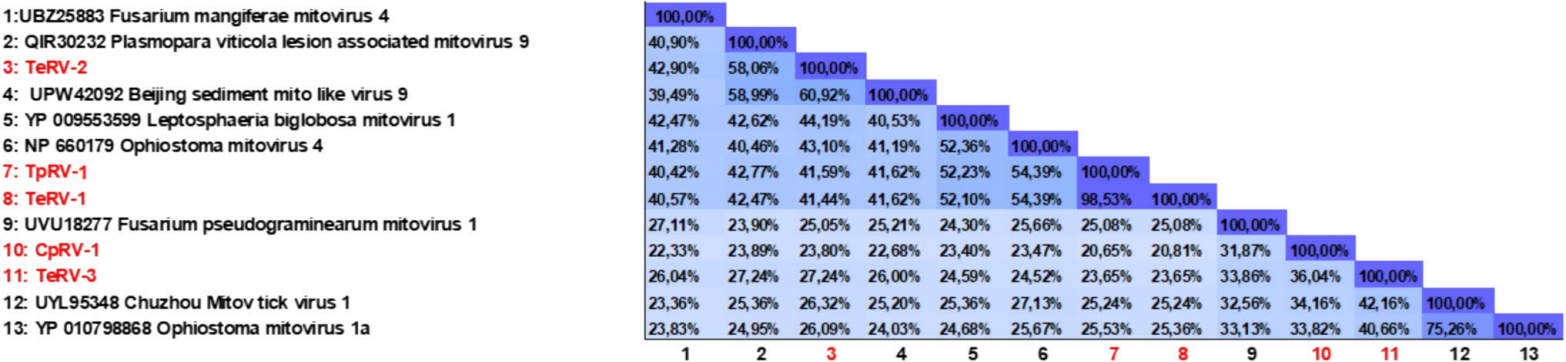
Percent Identity Matrix of the mitoviruses from this study, and other viral members from *Mitoviridae*, generated using Clustal Omega. The color gradients represent percent identities, with darker blue shades indicating higher identities and lighter shades indicating lower identities. The mitoviruses are numbered from 1 to 13, and their corresponding labels are displayed at the bottom of each column. Mitoviruses from this study are highlighted in red for better readability

Phylogenetic analysis revealed that TeRV-1, TeRV-2, and TpRV-1 clustered most closely with members of the genus *Unuamitovirus*, while CpRV-1 and TeRV-3 clustered with viruses from *Duamitovirus* (Fig. 1). The percent identities of the RdRps for CpRV-1, TeRV-3, and TeRV-2 (Fig. 2) in comparison to other mitoviruses were found to be below the designated threshold of 90%, which serves as the criterion for species demarcation within the *Mitoviridae* family [56]. This suggests the novel nature of these mycoviruses, classifying them within the genera *Duamitovirus* (CpRV-1 and TeRV-3) and *Unuamitovirus* (TeRV-1, TeRV-2, and TpRV-1). Notably, TeRV-1 and TpRV-1 variants exhibited a remarkable 98.53% identity to one another at the RdRp level, indicating that they likely to be members of the same putative species.

**Fig. 3:**
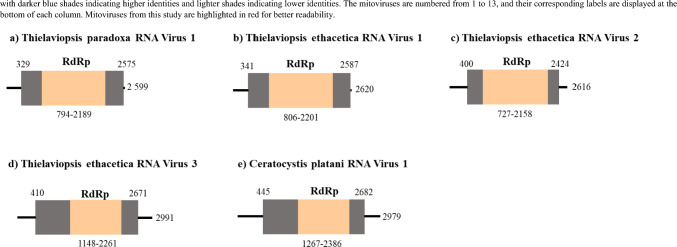
Schematic representation of the genomic organization of **a** TpRV-1, **b** TeRV-1, **c** TeRV-2, **d** TeRV-3, and **e** CpRV-1. The open bars represent single open reading frames (ORFs), while the single lines represent untranslated regions (UTRs). The length of the ORF is indicated for all mitoviruses, and the position of the RdRp domain encoded on the ORF is also shown

The genomes of all putative mitoviruses were characterized and a detailed diagrammatic representation of their genome organizations can be found in Fig. 3. All five putative mitoviral genomes were composed of a single ORF, which contained an RdRp protein domain.

The genome organizations, including the length of the ORF and all associated protein domains, of the unuamitoviruses TpRV-1, TeRV-1, and TeRV-2 is indicated in Fig. 3a, b, and c, respectively. The predicted mass of the protein product produced by ORFs of these viruses were approximately 87.53 kDa for both TpRV-1 and TeRV-1, and 78.23 kDa for TeRV-2. A Blastp analysis of the RdRp protein domain from TeRV-2 exhibited the highest percent identity to Beijing sediment mito-like virus 9 (61.47% identity, 98% coverage, 0.0 E-value), while those of TpRV-1 and TeRV-1 displayed the highest similarity to Ophiostoma mitovirus 4 (52.49% identity, 99% coverage, and 0.0 *E*-value). All unuamitoviruses displayed 5’ and 3’ UTRs of varying lengths, and the secondary structures, as well as the associated free energy of these were assessed and is indicated in Figs. S3, S4, and S5 for TpRV-1, TeRV-1, and TeRV-2, respectively. The analysis revealed the formation of multiple stem loop structures in all three mitoviruses, with free energy values promoting the formation of stable stem-loop structures. Additionally, the terminal ends of all three unuamitoviruses showed reverse complementarity, and may thus potentially form panhandle structures, albeit with varying free energy values. These are indicated in Fig. S3C (TpRV-1), Fig. S4C (TeRV-1), and Fig. S5C (TeRV-2).

The genome organizations of TeRV-3 and CpRV-1, putative members of the genus *Duamitovirus*, can be found in Fig. 3d, and e, respectively. The predicted protein mass for the protein product produced by the ORF of TeRV-3 was 86.75 kDa, and the RdRp when analyzed with Blastp displayed the highest percent identity to Guangdong mito-like virus 6 (43.19% identity, 95% coverage, 1e − 180 value). The remaining duamitovirus, CpRV-1, displayed a predicted protein mass of 85.78 kDa, and similarly to TeRV-3, also displayed an RdRp percent identity that was closer to that of the Guangdong mito-like virus 6 (39.00% identity, 74% coverage, 3e − 106 *E*-value). As with the unuamitoviruses from this study, both duamitoviruses contained 5’ and 3’ UTRs of varying lengths, which formed stable stem-loop structures with varying free energy values. These structures, as well as all associated energy values, are indicated in Fig. S6 for TeRV-3, and Fig. S7 for CpRV-1. Based on the complementarity of the 3’ and 5’ UTRs, and the predicted free energy values, both duamitoviruses may potentially form a panhandle structure, as indicated in Fig. S6C for TeRV-3 and Fig. S7C for CpRV-1.

Amino acid alignments of the RdRp sequences from all mitoviruses analyzed in this study, and other closely related viruses also revealed six conserved protein motifs (I to VI), typical of viral RdRps [55, 56] (Fig. S1). It should be noted however that, that an in-depth analysis of mitoviral protein motifs have also recently been performed, and has shown that there are five unique protein motifs typically conserved across *Mitoviridae* [57].

### Characterization of mycoviruses from totiviridae

Two putative mycoviruses with dsRNA genomes were found in the *H. omanensis* CMW 44450 and *T. paradoxa* transcriptomes, respectively. Notably, these mycoviruses exhibited significant similarity to viruses belonging to the family *Totiviridae*. The totivirus from *H. omanensis* CMW 44450 was provisionally named Huntiella omanensis RNA Virus 1 (HoRV-1). Two segments, potentially belonging to HoRV-1, was found in the transcriptome of this fungus and was named Huntiella omanensis RNA virus ORF 1 (HoRV1_ORF-1), and Huntiella omanensis RNA virus ORF2 (HoRV1_ORF2). The totivirus associating with *T. paradoxa* was tentatively named Thielaviopsis paradoxa RNA Virus 2 (TpRV-2).

**Fig. 4:**
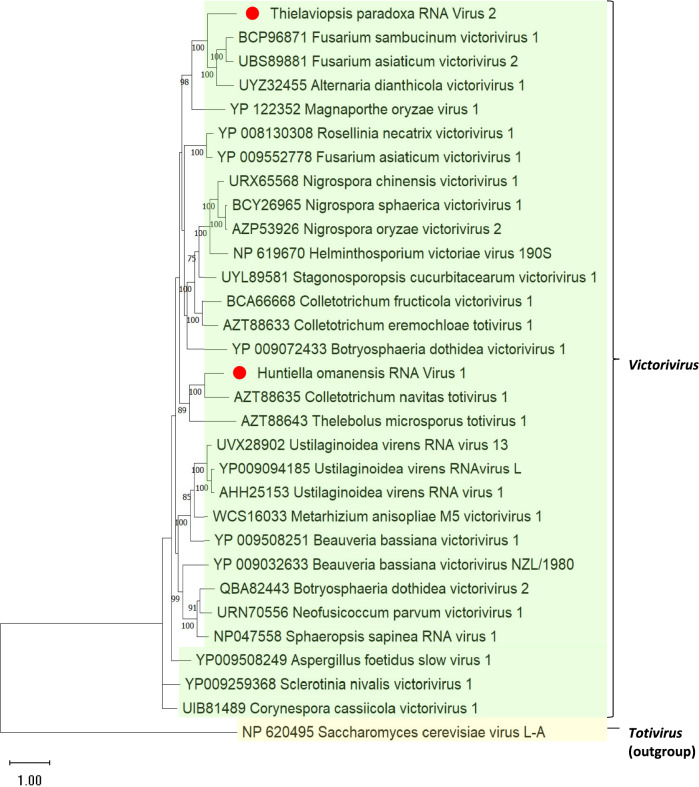
Maximum likelihood phylogenetic tree constructed from the alignment of RdRp protein sequences from totiviruses discovered in this study, with those of related viruses. The phylogeny was generated using IQ-TREE, using the Le Gauss model with frequencies, invariant sites, and gamma distribution. Bootstrapping with 1000 replicates was performed, and corresponding percentages are indicated next to the branches. Only bootstrap values equal to or exceeding 70 are displayed. The Saccharomyces cerevisiae virus L-A was used as an outgroup. The red circles (●) indicate the totiviruses discovered in this study

**Fig. 5:**
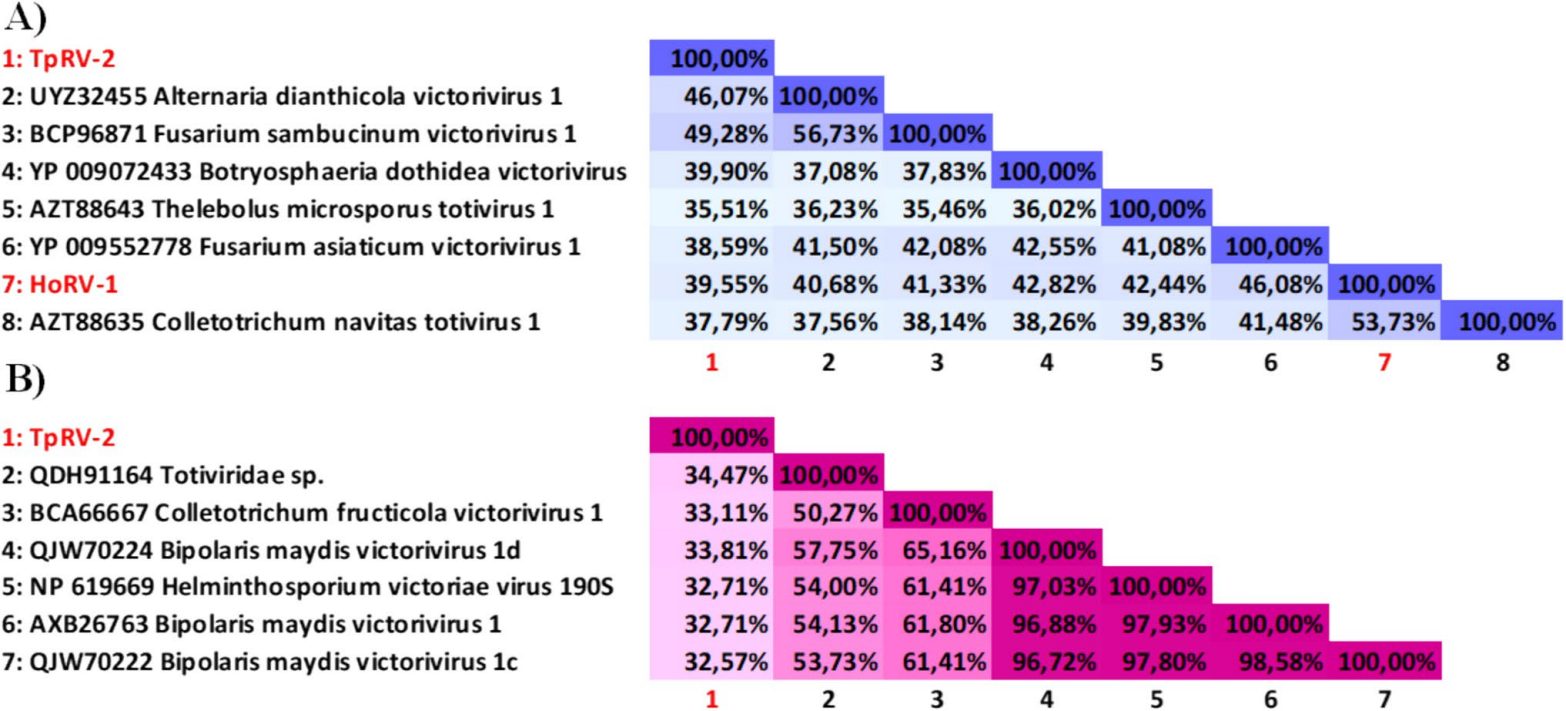
Percent Identity Matrix of the totiviruses from this study, and other viral members from the genus *Victorivirus*. The Matrix was generated using Clustal-Omega 1.2.2, with the color gradients serving to represent the respective percent identities of the totiviruses from this study to other related viruses. The percent identity matrixes are presented as A, denoting the alignments of the **a** RdRp and **b** those of the CP amino acid sequences. The percent identities are visualized with varying shades of blue and pink in the RdRp and CP alignments, respectively. Higher percentages are indicated in darker shades, while lower percentages are indicated in lighter hues. For clarity, the totiviruses from this study are numbered as 1–7 in the CP alignments and as 1–8 in the RdRp alignments. Corresponding labels for each of these totiviruses

To elucidate the relationships among the totiviruses, a phylogenetic tree was constructed using the amino acid sequences of the RdRps from TpRV-2 and HoRV-1, and cognate sequences derived from references obtained from GenBank. The resulting phylogenetic tree (Fig. 4) showed clustering of both TpRV-2 and HoRV-1 with viral members belonging to the genus *Victorivirus*. A comparative analysis of the RdRp and CP sequences of various totiviruses and closely related viruses was conducted, and percent identity matrices were constructed to delineate distinct viral species (Fig. 5). The findings revealed that the RdRp percent identities of HoRV-1 and TpRV-2 (Fig. 5a), in comparison to other totiviruses, fell below the species demarcation threshold of 60% [58]. Percent identities for the TpRV-2 CP sequences were also below 60% (Fig. 5b), further supporting its classification as a distinct species within the *Victorivirus* genus. It is worth noting that the segment HoRV1_ORF1 encoded for a truncated CP, consequently precluding the generation of a percent identity matrix for this particular protein and was excluded from further genomic characterization.

**Fig. 6:**
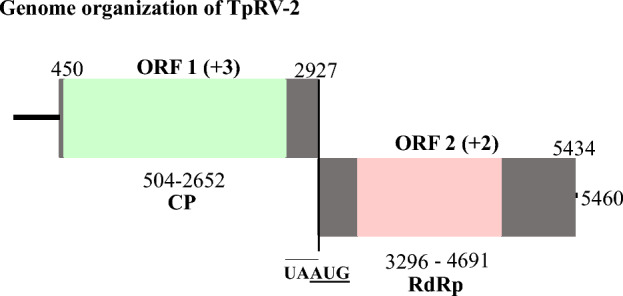
Schematic representation of the genome organization of TpRV-2. The open bars signify individual ORFs, and the single lines represent the 5’ and 3’ UTRs. The putative victorivirus consists of two ORFs, and their respective lengths are indicated. Moreover, the positions of the RdRp and CP domains encoded within their corresponding ORFs are shown. The reading frames for each ORF are clearly indicated, and the stop-initiation codon required for the translation of ORF 2 is also depicted in the figure

TpRV-2 had a genome consisting of a complete totivirus CP domain and another ORF encoding for a complete RdRp protein domain. The genome organization of TpRV-2, as well as the length of all ORFs and protein domains, can be found in Fig. 6. Amino acid alignments of the RdRp sequences from HoRV-1, TpRV-2 and other closely related viruses also revealed eight conserved protein motifs (I–VIII), typical of victorivirus RdRps (Fig. S2). The genome of TpRV-2 consists of a 5’ and 3’ UTR, as well as two overlapping ORFs. The first ORF, which is in the + 3-reading frame, encodes for a CP, with a predicted molecular mass of 86.59 kDa. This protein, when analyzed with Blastp, was found to exhibit significant sequence similarity to the virus termed ‘Totiviridae sp.’ (35.28% identity, 80% coverage, 9e-93 E-value). The C-terminal ends of the TpRV-2 protein also lacks an Ala/Gly/Pro rich region which is sometimes present in the ORF encoding the CP in victoriviruses. ORF 1 and 2 overlaps with a stop-initiation codon (UAAUG), and a pseudoknot is predicted to span in this region, as indicated by Fig. S8. ORF 2, which is in the + 2-reading frame, encodes for an RdRp protein with a predicted mass of 91.72 kDa. This RdRp also has a higher percent identity to Fusarium sambucinum victorivirus 1 (49.64% identity, 98% coverage, 0.0 *E*-value).

## Discussion

The current study demonstrates the efficacy of utilizing metatranscriptomic approaches to investigate fungal transcriptomes, including those retrieved from publicly available repositories, for the discovery of mycoviruses. This method has been instrumental in the successful identification of numerous mycoviruses in other studies [34, 37, 38], and has resulted in the discovery of six novel mycoviruses in four fungal transcriptomes from Ceratocystidaceae in this study.

Mycoviruses with ( +) ssRNA genomes were more prevalent in this study when compared to those with dsRNA genomes. In the past, mycoviruses with ( +) ssRNA genomes were less frequently detected than mycoviruses with dsRNA genomes. This disparity can be attributed to the conventional culture-based methods used at the time, which relied on the purification of dsRNA or a dsRNA intermediate [59]. These techniques were less sensitive to the detection of ssRNA viruses, as the recovery of intermediate dsRNA (the replicative intermediate of ssRNA viruses) was less efficient than that of genomic dsRNA, leading to an under representation of many ssRNA mycoviruses [60, 61]. The findings from this study suggests that ( +) ssRNA viruses may be more prevalent in the fungal isolates evaluated in this study and underscore the suitability of employing transcriptome analysis for the discovery of ssRNA viruses.

The ( +) ssRNA mycoviruses showed similarity to members of the viral family *Mitoviridae*. *Mitoviridae* has recently earned recognition as a distinct family by the ICTV, which has led to the formation of 4 new genera (https://ictv.global/taxonomy). Mitoviruses generally possess genomes ranging in size from 2.0 to 4.5 Kb and are characterized by a single ORF that encodes an RdRp protein [62–65], consistent with the four mitoviruses from this study. Furthermore, alignments of the RdRp protein sequence of the mitoviruses from this study against those of other mitoviruses also revealed the presence of 6 protein motifs, which included a well conserved Glycine-Aspartic Acid-Aspartic Acid (GDD) motif. These were consistent with the six protein motifs which are commonly conserved among viruses belonging to *Mitoviridae* [62, 65–67].

Another unique feature of mitoviruses is their ability to utilize the mitochondrial genetic code during replication, where the codon UGA, typically a stop codon in the standard genetic code, also encodes for the amino acid tryptophan [68]. It should be noted however, that some mitoviruses do not contain an internal UGA codon and are hypothesized to have a similar cytoplasmic location to that of narnaviruses, or have hosts witch also have no or few UGA codons in their core mitochondrial genes [69]. Alternatively, their hosts may exhibit an absence or a limited presence of UGA codons in their core mitochondrial genes [70]. Nevertheless, the addition of the codon UGA in the genomes of most mitoviruses have allowed them to adapt to environmental conditions in the mitochondria of their fungal hosts [71]. The findings from this study supports this notion since the genomes of the identified mitoviruses only revealed complete ORFs when the mitochondrial genetic code was applied for ORF prediction.

Another characteristic commonly exhibited by the 3’ and 5’ UTRs of mitoviruses, is the formation of stable stem-loop structures, which can potentially form a panhandle structure through inverted complementarity [67, 72–74]. All the mitoviruses which were analyzed in this study were found to have stable stem-loop structures in their UTRs, suggesting the conservation of this genomic feature across the identified mycoviruses, as well as suggesting that the contigs represent complete genomes. These secondary structures have been hypothesized to play important roles in replication and translation of the mitoviruses and may also act as protective elements against degradation [75, 76]. However, it is important to note that a limitation of this study is the lack of cultures for the fungal isolates under investigation. As a result, it remains uncertain whether the UTRs of all viruses under investigation were complete, as 3’ and 5’ Rapid Amplification of cDNA Ends (RACE) PCRs, which are normally used to elucidate the terminal ends of the viral genome, could not be performed.

Phylogenetic analysis of all the mitoviruses from this study revealed that these viruses belonged to two established genera in the family *Mitoviridae*, namely *Unuamitovirus* and *Duamitovirus*. The criteria for assigning viruses to these genera are primarily based on their phylogenetic relationships, as official guidelines for taxonomic classification are currently lacking. Species demarcation within this family, however, dictates that the amino acid sequence identities of RdRp proteins among any putative mitoviruses should be below 90% when compared to other closely related viruses [56]. The mitoviruses TeRV-3 and CpRV-1 meet this criterion and are thus novel mycoviruses which may potentially belong to the genus *Duamitovirus*.

Despite having RdRp percent identities lower than 90% when compared to closely affiliated mitoviruses within the *Unuamitovirus* genus, the percent identity matrix of TpRV-1 and TeRV-1 revealed that these viruses exhibited a 98% identity to each other. This striking similarity indicates that TpRV-1 and TeRV-1 represents the same virus. The *T. ethacetica* and *T. paradoxa* isolates were isolated from Brazil (Sertãozinho) and Colombia (Bogotá), respectively. These species are known to infect some of the same hosts, including sugarcane [77, 78] and oil palm [79, 80]. This suggests that these fungal hosts might have come into contact at some point, potentially leading to the exchange of the mitoviruses. Interestingly, cases have been documented where different fungal species are naturally infected by the same mycovirus [81]. This suggests that certain mycoviruses can propagate between fungi within the same genus or even the same family. Various studies have also demonstrated that cross-kingdom transfer, where a fungal virus could be transmitted to a plant host and subsequently to another fungal host, may also facilitate the spread of mycoviruses [82, 83]. Therefore, TRV-1 might have spread between these two fungal isolates through a yet unidentified mechanism. Further exploration into the mechanisms underpinning the transfer of the virus between *T. ethacetica* and *T. paradoxa* is warranted to fully elucidate this phenomenon.

In this study, several mycoviral genomes exhibited significant truncation, which may be attributable to the library preparation method, which entailed a poly-A selection step selecting for RNA molecules with poly-A tails (mRNA-seq) [84]. Mycoviruses often lack poly-A tails, which may reduce their representation in such datasets [85–87]. The incorporation of a ribo-depletion step during library preparation may address this challenge by enriching non-ribosomal RNA [84]. An alternative explanation is that the truncated mycoviral contigs might have been present in low abundance within the fungal host, resulting in insufficient genome coverage during sequencing. The study unveiled the presence of truncated mycoviruses consisting of dsRNA genomes which exhibited homology to existing members of the viral family *Totiviridae*. These viruses, including HoRV1, had truncated genomes below the typical 4.6 to 7.0 kb range of *Totiviridae* members [11]. Notably, the choice of sequencing platform used for *H. omanensis*, Ion Torrent Proton, could also impact assembly due to inherent limitations, such as sequence errors linked to homopolymers [88]. These errors can cause insertions or deletions, leading to truncated contigs that do not represent the full viral genome [89]. The partial genome segments from HoRV-1, namely HoRV1_ORF1 and HoRV1_ORF2 each featured a single ORF, encoding an RdRp and a CP, respectively. Typically, totivirus genomes are non-segmented, and encodes for these protein domains in two separate ORFs [11]. Thus, the viral segments from HoRV-1 were not assembled into a complete genome. The separation of the two ORFs might be attributed to the influence of complex secondary structures, such as a pseudoknot situated upstream of the RdRp containing ORF, which may impede the reverse transcription step during library preparation [90]. It is essential to acknowledge however, that the coverage for these genomes was relatively low. Therefore, it's also possible that the assembler could not bridge the gap between these two contigs due to insufficient data. Despite the absence of an ORF encoding for a complete CP, HoRV-1 was included in phylogenetic analysis due the presence of a complete ORF encoding for an RdRp domain and was subsequently found to cluster with members of the genus *Victorivirus*.

The remaining totivirus, TpRV-2, conformed to the expected genome size and organization of members within the *Totiviridae* family. Furthermore, phylogenetic analysis revealed that TpRV-2 clustered with members of the genus *Victorivirus*. The genome of this virus also contained an H-type pseudoknot structure upstream of the RdRp encoding ORF and contains an UAAUG overlap region like other members of this genus [91–93]. The demarcation criteria for species within this genus necessitate an amino acid sequence identity percentage of less than 60% with other closely related viruses at the RdRp and Cp protein level [58]. Since TpRV-2 meets these requirements, it is likely a novel member of the genus *Victorivirus*.

Overall, this study enhances our understanding of mycoviral diversity within the fungal family Ceratocystidaceae. However, it should be noted that this study's scope was limited to publicly available transcriptomes, which resulted in the analysis of only a subset of species within the Ceratocystidaceae family. To gain a more comprehensive understanding of mycoviral diversity, acquiring additional sequencing data for a broader range of family members would be advantageous. Nevertheless, the present study has identified novel mycoviruses in four additional species from this family, namely *C. platani*, *H. omanensis*, *T. paradoxa,* and *T. ethacetica*. Notably, *C. platani*, *T. paradoxa*, and *T. ethacetica* are significant fungal pathogens of plants, known for their detrimental impact on economically important crops and trees [40, 77, 80, 94–97]. Several studies have shown that mycoviruses show potential as biocontrol agents against plant pathogenic fungi, due to the ability of some to induce hypovirulence in the host [98–101]. Therefore, the identification of mycoviruses within these fungal species may contribute to the future development of biocontrol strategies against them. Interestingly, the majority of mycoviruses that were present in these genera were mitoviruses. Several studies have shown that some mitoviruses possess the capability to induce hypovirulence in their plant pathogenic hosts [76, 102, 103]. Research by Shackelton and Holmes [104] suggests that hypovirulent mitoviruses might have originated from plant hosts, which deployed them as a strategic defense mechanism against invasive fungal threats. Studies have also characterized hypovirulence-inducing mycoviruses in the families *Totiviridae* and *Endornaviridae* [105–108]. It is thus possible that the mycoviruses from this study may also confer hypovirulence to their plant pathogenic fungal hosts, although this will have to be evaluated further.

## Conclusion

In summary, this investigation has enhanced our knowledge of the mycoviral landscape within the relatively unexplored fungal family Ceratocystidaceae. The study marked the discovery of the first mycoviruses in several fungal species, including *C. platani, T. ethacetica*, *T. paradoxa*, and *H. omanensis*. The utilization of fungal transcriptomes from publicly available databases proved to be a valuable approach, resulting in the analysis of 10 fungal transcriptomes and the identification of six novel mycoviruses. These were primarily ( +) ssRNA viruses, mainly belonging to the *Mitoviridae* family. Additionally, three mycoviruses with dsRNA genomes from the *Totiviridae* family were identified, with phylogenetic analysis conducted for two of these. However, limitations included restricted transcriptomic datasets, the utilization of mRNA-seq for the majority of fungal isolates evaluated, and the need for further molecular techniques to confirm and explore these mycoviruses in their original hosts. Future research should assess the impact of these mycoviruses on their fungal hosts and consider their potential as biocontrol agents for the fungal isolates examined in this study.

## Supplementary information

Below is the link to the electronic supplementary material.

Supplementary file1 (PDF 388 KB)

Supplementary file2 (PDF 688 KB)

Supplementary file3 (PDF 125 KB)

Supplementary file4 (TXT 8 KB)

Supplementary file5 (TXT 6 KB)

Supplementary file6 (TXT 7 KB)

Supplementary file7 (TXT 7 KB)

Supplementary file8 (TXT 7 KB)

Supplementary file9 (TXT 8 KB)

Supplementary file10 (TXT 7 KB)

## Data Availability

The transcriptomes used in the identification of viral sequences in this study are publicly available on the National Center for Biotechnology Information Sequencing Reads Archive. The accession numbers for these are indicated in Table 1, provided within the manuscript. The sequence data of all viruses identified this study have been deposited in the National Center for Biotechnology Information with the primary accession codes BK065014-BK065021.
