# Supplementary material for: Identification and characterization of mycoviruses in transcriptomes from the fungal family ceratocystidaceae
Source: Virus Genes. 2024 Oct 8;60(6):696–710. doi: 10.1007/s11262-024-02112-4 (PMC11568016; doi:10.1007/s11262-024-02112-4)
Supplement: Supplementary file 1 — Supplementary file1 (PDF 388 KB) [file 11262_2024_2112_MOESM1_ESM.pdf]

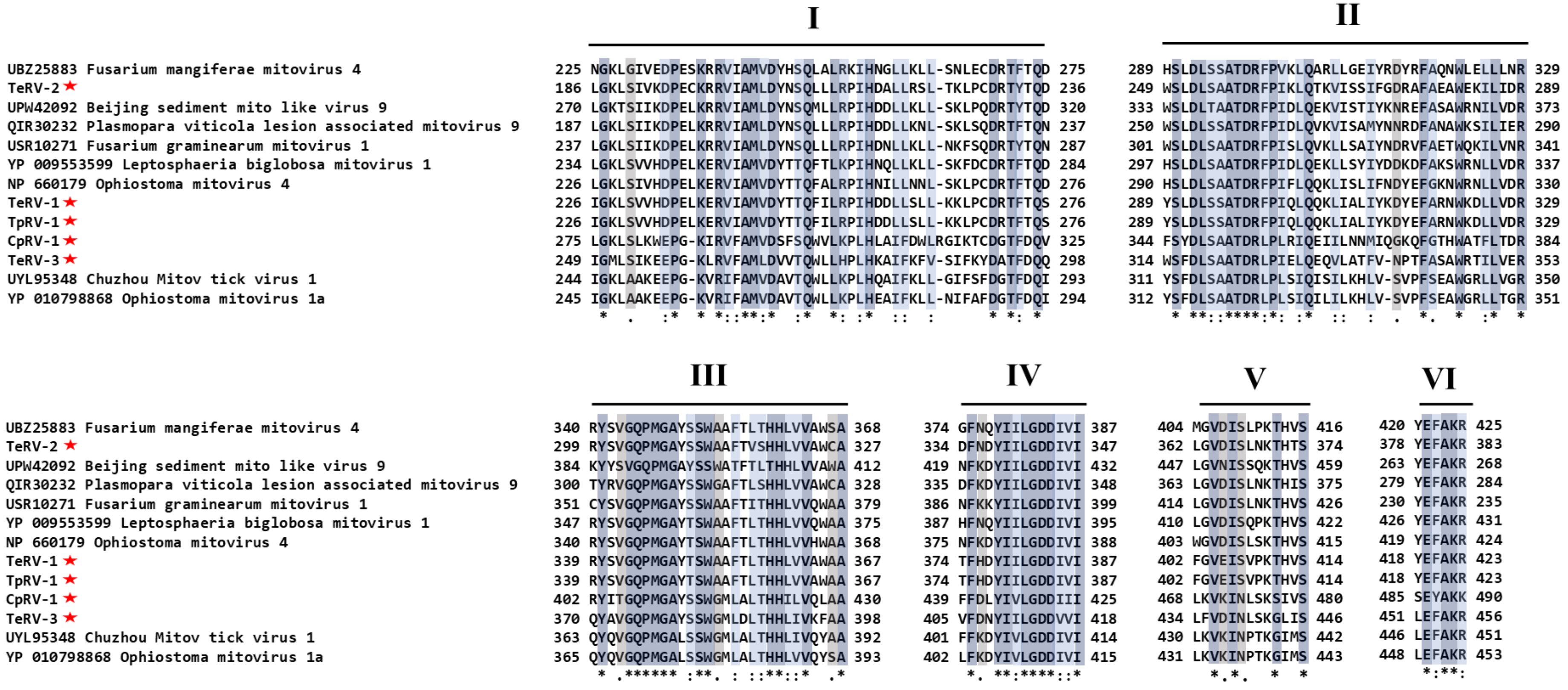

Fig. S1 Multiple sequence alignment of the RdRp amino acid sequences from the mitoviruses investigated in this study (Indicated by the red stars), and other selected viruses from Mitoviridae. The alignment was generated using Clustal Omega 1.2.1 with default parameters. Conserved residues are denoted by an asterisk (*) and are highlighted in dark blue. Colons (:) and dots (.) signify conserved and semiconserved residues, respectively, and are coloured light blue and grey. There were 6 conserved motifs in total, and these are numbered from I to VI.

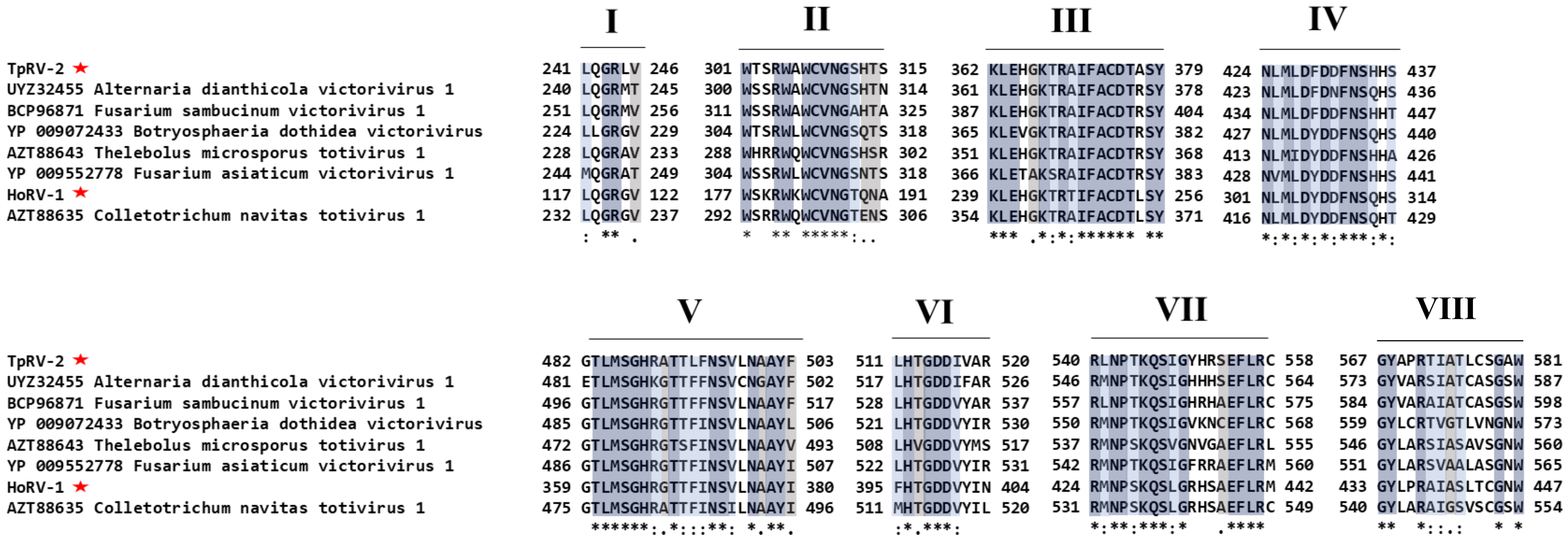

Fig. S2 Multiple sequence alignment of the RdRp amino acid Sequences from the totiviruses investigated in this study (Indicated by the red stars), and other selected viruses from the genus Victorivirus. The alignment was generated using Clustal Omega 1.2.1 with default parameters. Conserved residues are denoted by an asterisk (*) and are highlighted in dark blue. Colons (:) and dots (.) signify conserved and semi-conserved residues, respectively, and are coloured light blue and grey. There were 8 conserved motifs in total, and these are numbered from I to VIII.
