# Supplementary material for: Identification and characterization of mycoviruses in transcriptomes from the fungal family ceratocystidaceae
Source: Virus Genes. 2024 Oct 8;60(6):696–710. doi: 10.1007/s11262-024-02112-4 (PMC11568016; doi:10.1007/s11262-024-02112-4)
Supplement: Supplementary file 2 — Supplementary file2 (PDF 688 KB) [file 11262_2024_2112_MOESM2_ESM.pdf]

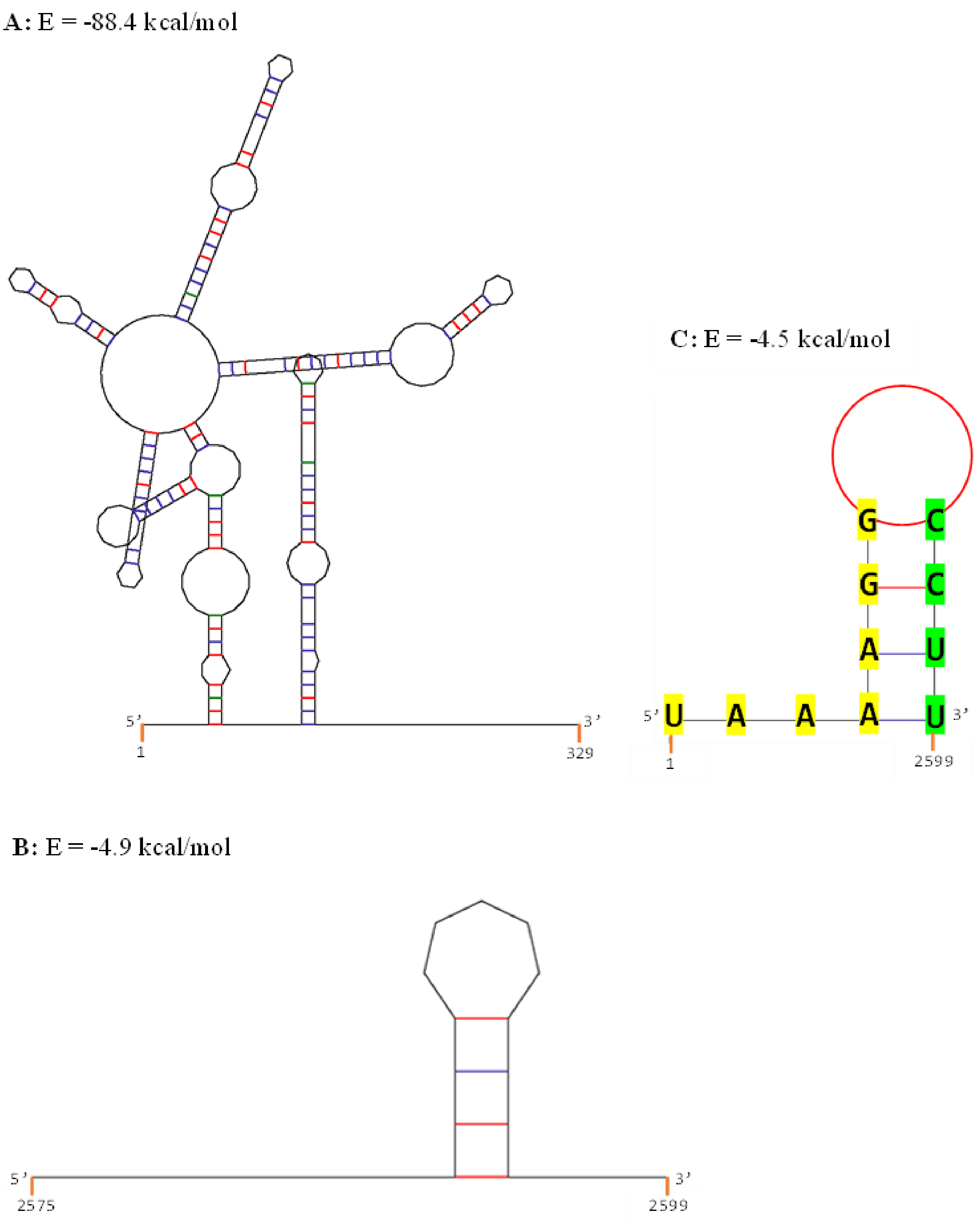

Fig. S3 Predicted secondary structures of the 5' (A) and 3' (B) terminal UTRs of TpRV-1, as well as the predicted panhandle structure (C) formed through complementary pairing between the two UTRs. Nucleotide positions 1 and 2599 represent the first and last nucleotide in the 5' and 3' UTRs, respectively. In the panhandle structure, complementary bases between sections of the 5' and 3' UTRs are highlighted: yellow for the 5' UTR and green for the 3' UTR. For the 3' UTR, complementary base pairing starts at position 2596, and ends at position 2599. Complementary base pairing for the 5' UTR starts at position 4 and ends at position 7. The red circle (not drawn to scale) represents the inner RNA Molecule. The secondary structures were predicted using Mfold.

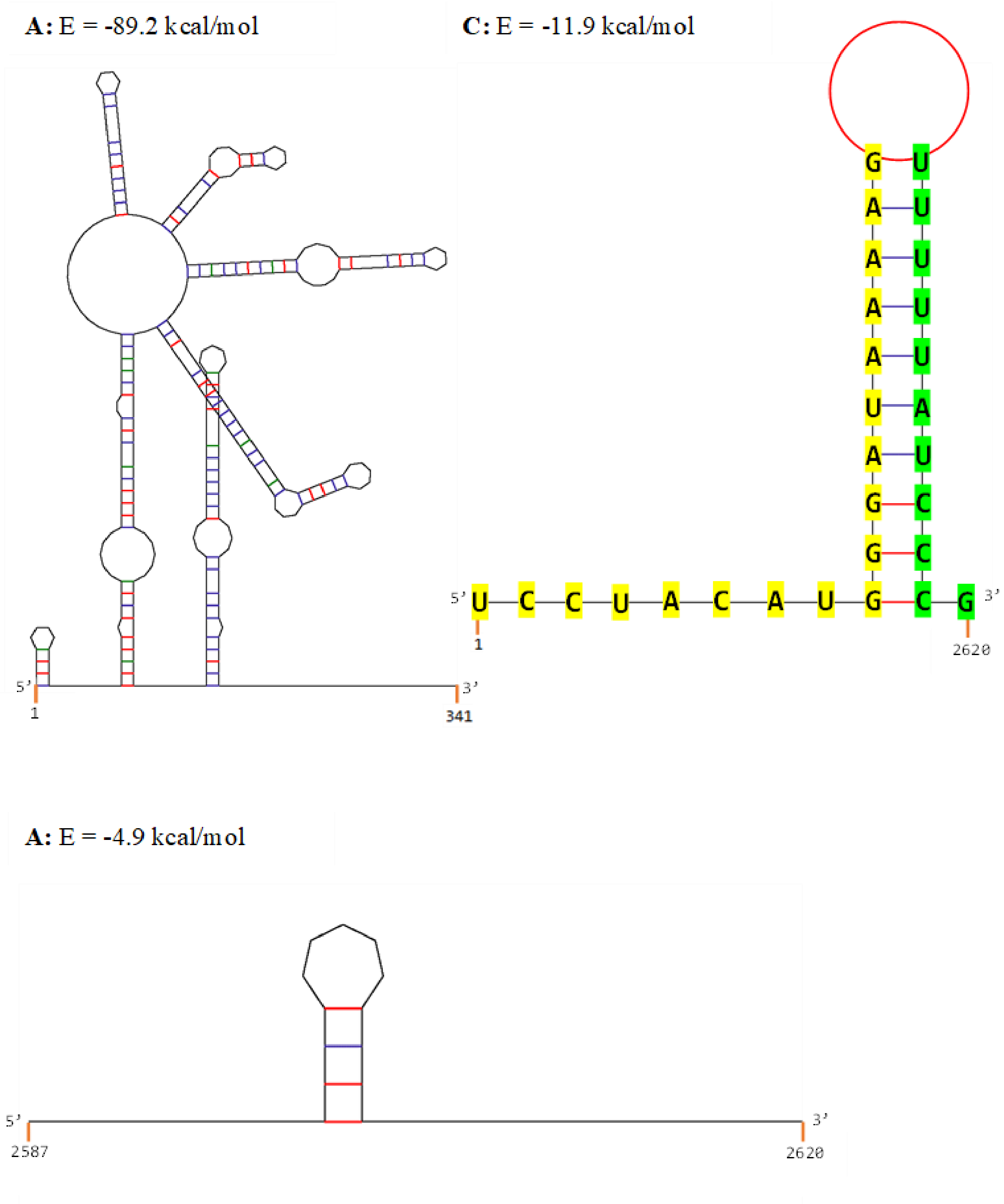

Fig. S4 Predicted secondary structures of the 5’ (A) and 3’ (B) terminal UTRs of TeRV-1, as well as the predicted panhandle structure (C) formed through complementary pairing between the two UTRs. Nucleotide positions 1 and 2620 represent the first and last nucleotide in the 5’ and 3’ UTRs, respectively. In the panhandle structure, complementary bases between sections of the 5' and 3' UTRs are highlighted: yellow for the 5' UTR and green for the 3' UTR. For the 3’ UTR, complementary base pairing starts at position 2610, and ends at position 2619. Complementary base pairing for the 5’ UTR starts at position 9 and ends at position 18. The red circle (not drawn to scale) represents the inner RNA Molecule. The secondary structures were predicted using Mfold.

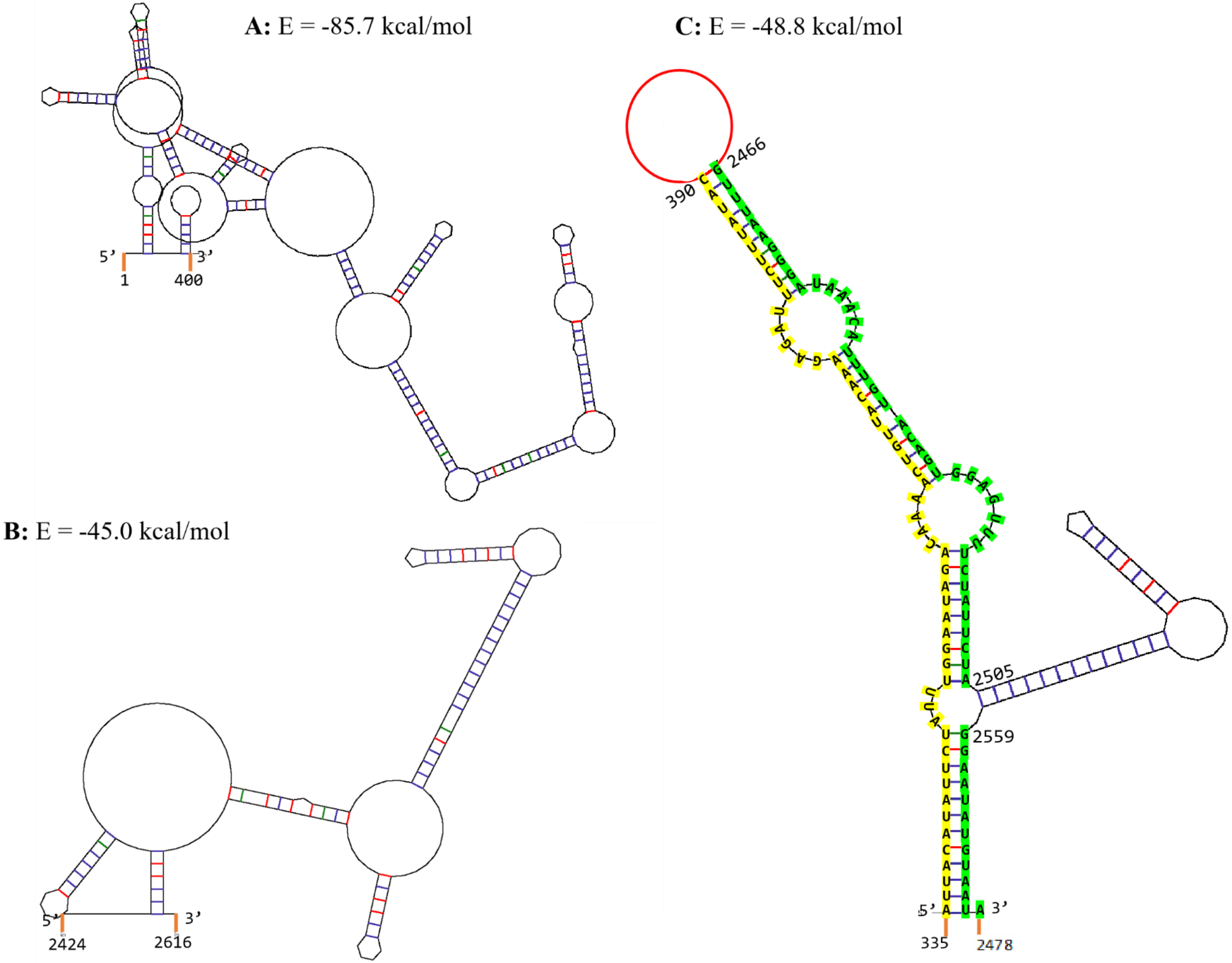

Fig. S5 Predicted secondary structures of the TeRV-2 5' (A) and 3' (B) terminal UTRs, as well as the predicted panhandle structure (C) formed through complementary pairing between the two UTRs. In the panhandle structure, complementary bases between sections of the 5' and 3' UTRs are highlighted: yellow for the 5' UTR and green for the 3' UTR. Nucleotide position 1 and 2616 represent the first and last nucleotide in the 5' and 3' UTRs, respectively. For the 3' UTR, complementary base pairing starts at position 2466, and ends at position 2505. This is followed by self-complementarity and the formation of secondary structures of the 3' terminal end, which ends at position 2559. Complementary base pairing between the 3' UTR and the 5' UTR then continues and ends at position 2477. Complementary base pairing for the 5' UTR starts at position 335 and ends at position 390. The red circle (not drawn to scale) represents the rest of the inner RA molecule. The secondary structures were predicted using Mfold.

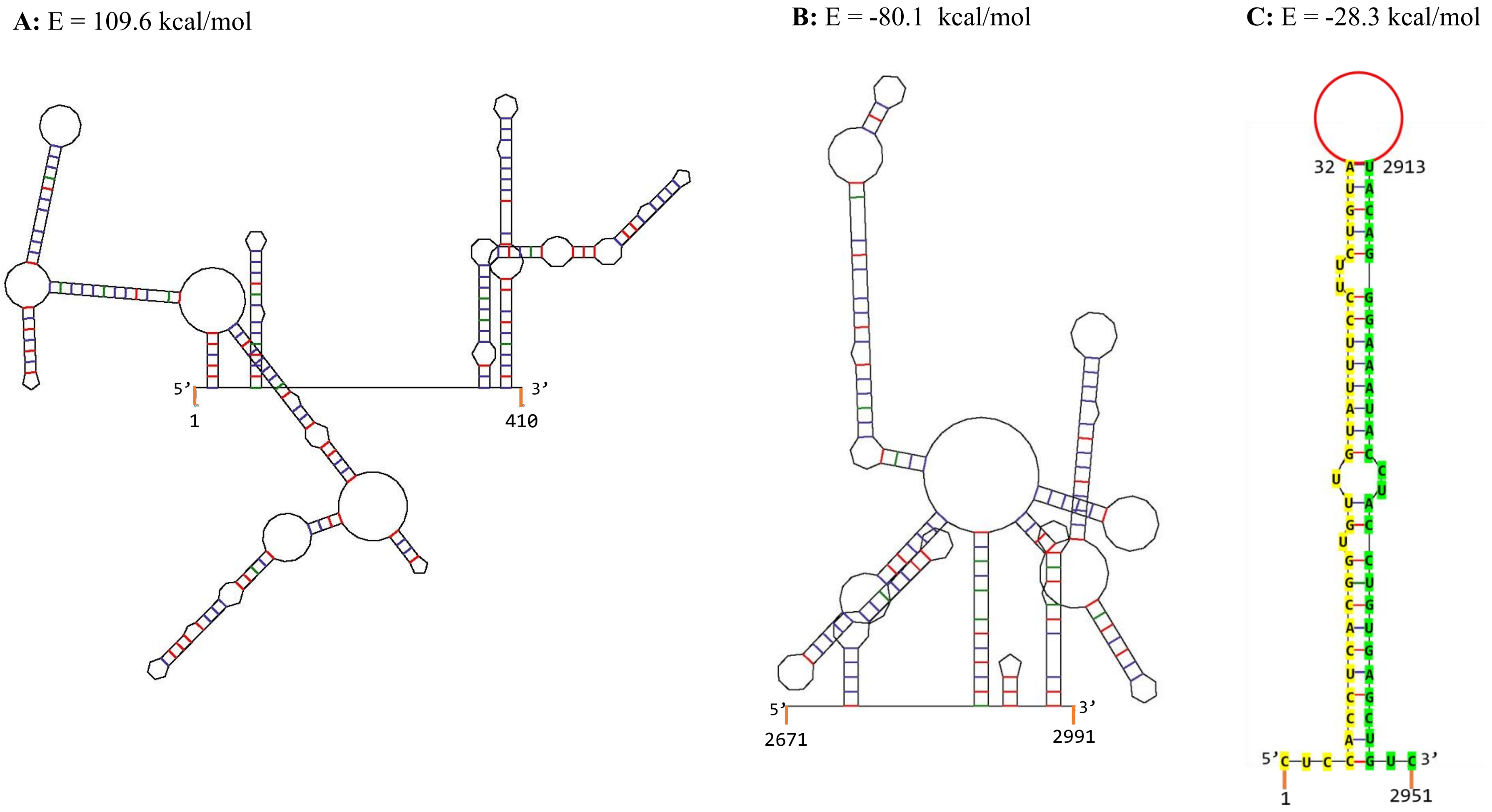

Fig. S6 Predicted secondary structures of the TeRV-3 5' (A) and 3' (B) terminal UTRs, as well as the predicted panhandle structure (C) formed through complementary pairing between the two UTRs. In the panhandle structure, complementary bases between sections of the 5' and 3' UTRs are highlighted: yellow for the 5' UTR and green for the 3' UTR. Nucleotide positions 1 and 2991 represent the first and last nucleotide in the 5' and 3' UTRs, respectively. For the 3' UTR, complementary base pairing starts at position 2913, and ends at position 2949. Complementary base pairing for the 5' UTR starts at position 4 and ends at position 32. The red circle (not drawn to scale) represents the rest of the inner RNA molecule. The secondary structures were predicted using Mfold.

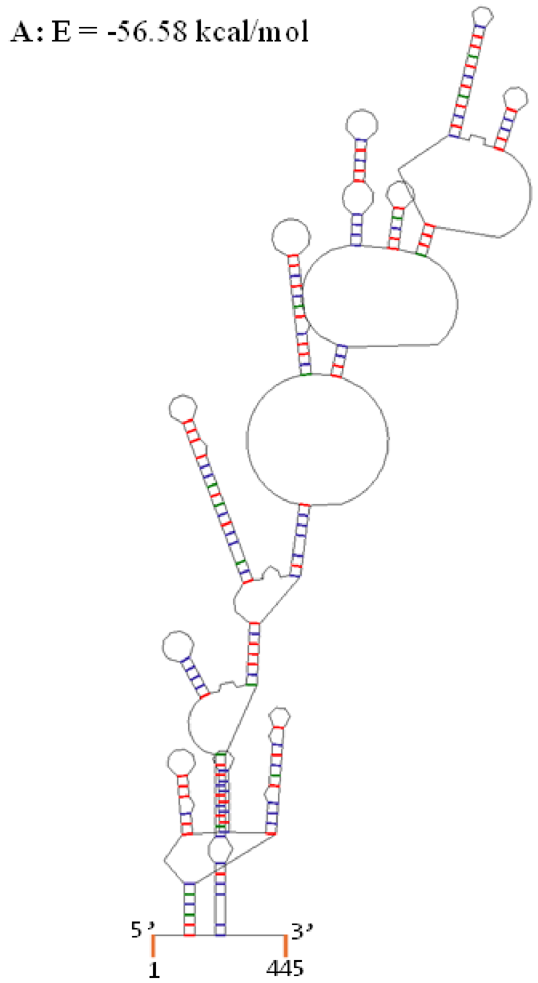

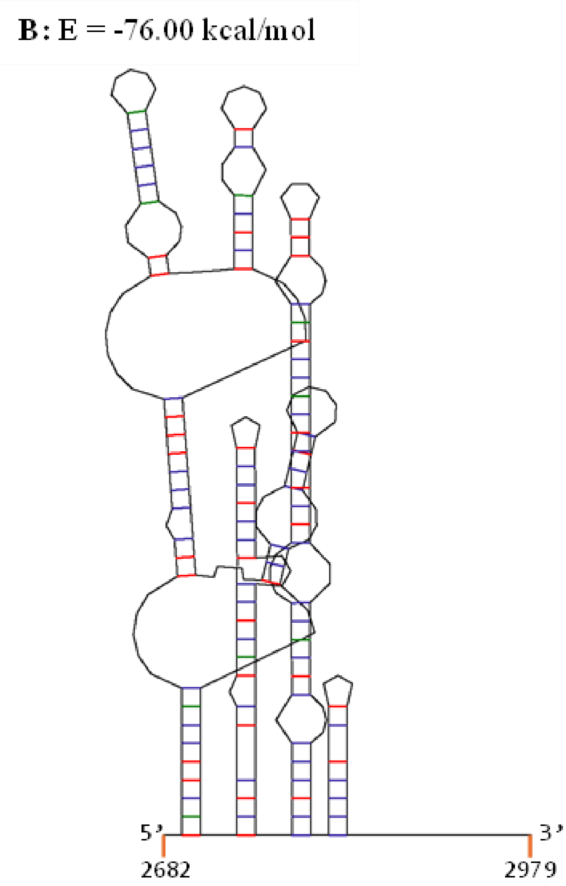

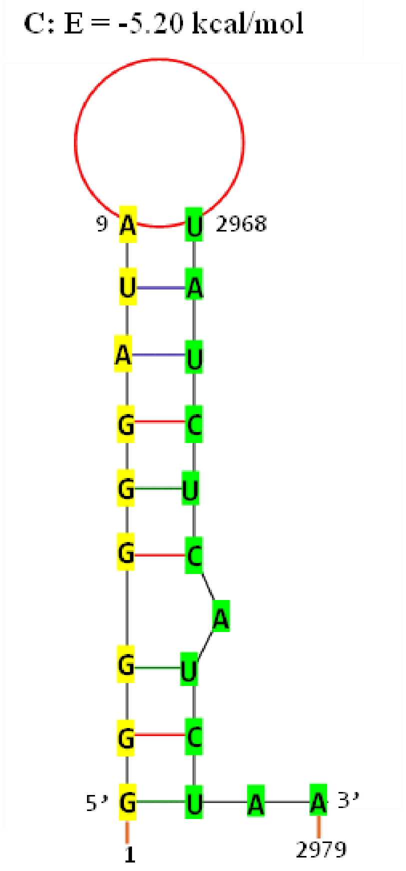

Fig. S7 Predicted secondary structures of the CpRV-1 5' (A) and 3' (B) terminal UTRs, as well as the predicted panhandle structure (C) formed through complementary pairing between the two UTRs. In the panhandle structure, complementary bases between sections of the 5' and 3' UTRs are highlighted: yellow for the 5' UTR and green for the 3' UTR. Nucleotide positions 1 and 2979 represent the first and last nucleotide in the 5' and 3' UTRs, respectively. For the 3' UTR, complementary base pairing starts at position 2976, and ends at position 2968. Complementary base pairing for the 5' UTR starts at position 1 and ends at position 9. The red circle (not drawn to scale) represents the rest of the inner RNA molecule. The secondary structures were predicted using Mfold.
