# Supplementary material for: Identification and characterization of mycoviruses in transcriptomes from the fungal family ceratocystidaceae
Source: Virus Genes. 2024 Oct 8;60(6):696–710. doi: 10.1007/s11262-024-02112-4 (PMC11568016; doi:10.1007/s11262-024-02112-4)
Supplement: Supplementary file 3 — Supplementary file3 (PDF 125 KB) [file 11262_2024_2112_MOESM3_ESM.pdf]

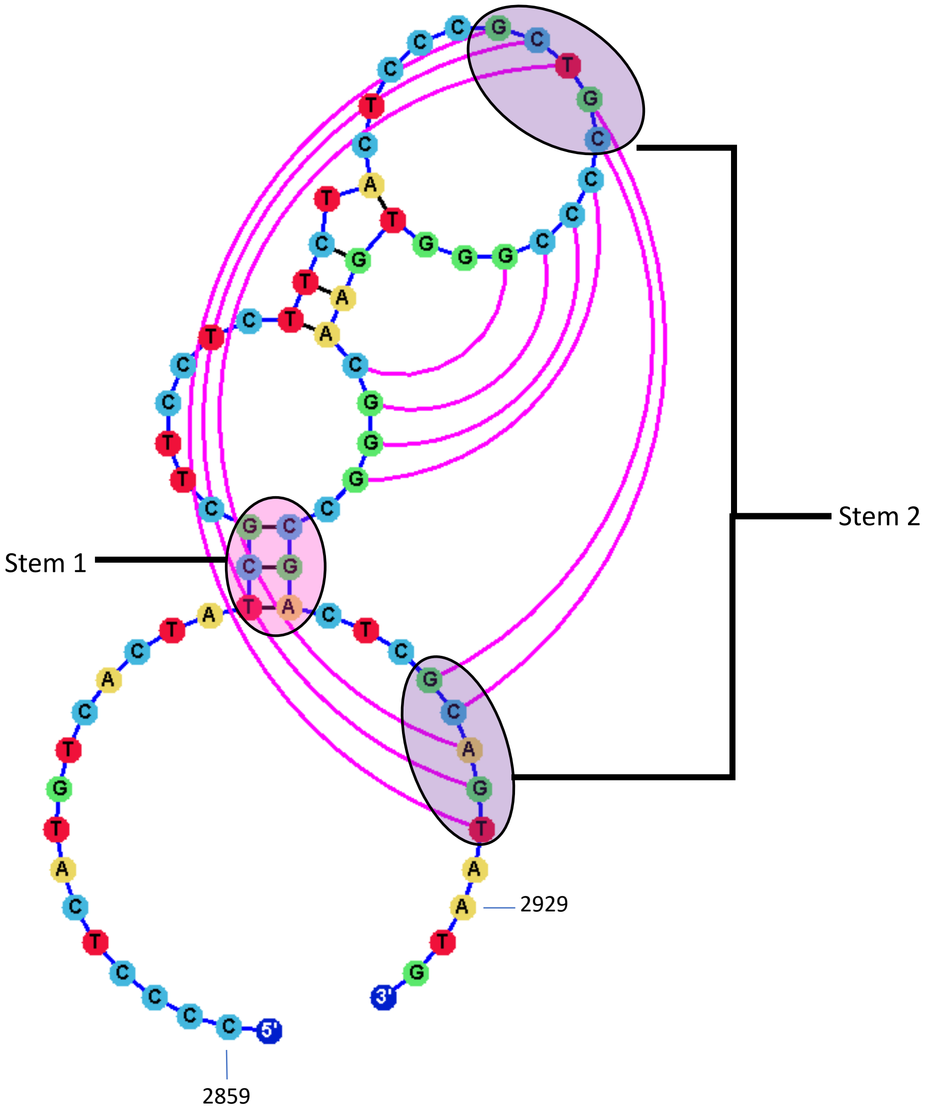

Fig S8. Predicted pseudoknot structure spanning the stop-start region of TpRV-2. The nucleotide positions are indicated. Stem 1 and Stem 2 are indicated by purple and pink ovals, respectively. Structures were predicted with ProbKnot and visualized with jViz.RNA 4.0.
